# Does COVID-19 infection increase the risk of pressure injury in critically ill patients? A narrative review

**DOI:** 10.1097/MD.0000000000029058

**Published:** 2022-03-18

**Authors:** Miriam Viviane Baron, Michele Paula dos Santos, Taís Michele Werle, Gabriela D.L.G. Scherer, Mariana Martins Dantas Santos, Luis Manuel Ley Dominguez, Cristine Brandenburg, Gabriela Feltez, Aline Ronis Sampaio, Marcus Vinicius de Mello Pinto, Sonia Carvalho, Patrícia Froes Meyer, Felice Picariello, Esteban Fortuny Pacheco, Isabel Cristina Reinheimer, Alexandre Gomes Sancho, Bartira Ercília Pinheiro da Costa

**Affiliations:** ^a^ *Graduate Program in Medicine and Health Sciences of the Pontifical Catholic University of Rio Grande do Sul (PUC/RS), Porto Alegre, Rio Grande do Sul, Brazil,* ^b^ *School of Medicine of the Pontifical Catholic University of Rio Grande do Sul (PUC/RS), Porto Alegre, Rio Grande do Sul, Brazil,* ^c^ *Universidad Popular Autónoma del Estado de Puebla, Puebla, Puebla, México,* ^d^ *Universidade Estadual Vale do Acaraú, Fortaleza, Ceará, Brazil,* ^e^ *Federal University of Health Sciences of Porto Alegre, Porto Alegre, Rio Grande do Sul, Brazil,* ^f^ *Instituto Celulare, Itaipava, Rio de Janeiro, Brazil,* ^g^ *Rigshospital, Copenhagen East, København, Denmark,* ^h^ *Centro Universitário do Rio Grande do Norte, Natal, Rio Grande do Norte, Brazil,* ^i^ *Università degli Studi di Napoli Federico II, Napoli, Campania, Italia,* ^j^ *Universidad Finis Terrae, Providencia, Santiago, Chile,* ^k^ *Unigranrio, Rio de Janeiro, Rio de Janeiro, Brazil.*

**Keywords:** blood clotting, cytokines, ischemia, pressure ulcer, SARS-CoV-2

## Abstract

Patients with severe COVID-19 may have endothelial dysfunction and a hypercoagulable state that can cause skin damage. In the presence of external pressure on the tissues, the local inflammatory process regulated by inflammatory cytokines can increase and prolong itself, contributing to the formation of pressure injury (PI). PI is defined as localized damage to the skin or underlying tissues. It usually occurs as a result of intense and/or prolonged pressure in combination with shear. The aim of the study is to perform a narrative review on the physiological evidence of increased risk in the development of PI in critically ill patients with COVID-19.

In patients with severe COVID-19 a pattern of tissue damage consistent with complement-mediated microvascular injury was found in the lungs and skin of critically ill COVID-19 patients, suggesting sustained systemic activation of complement pathways. Theoretically, the same thrombogenic vascular changes related to COVID-19 that occur in the skin also occur in the underlying tissues, making patients less tolerant to the harmful effects of pressure and shear. Unlike the syndromes typical of acute respiratory illnesses and other pathologies that commonly lead to intensive care unit admission, COVID-19 and systemic viral spread show that local and systemic factors overlap. This fact may be justified by current epidemiological data showing that the prevalence of PI among intensive care unit patients with COVID-19 was 3 times higher than in those without COVID-19. This narrative review presents physiological evidence to suggesting an increased risk of developing PI in critically ill patients with COVID-19.

## 1. Introduction

In December 2019, the etiologic agent of severe acute respiratory syndrome-related coronavirus (SARS-CoV) -2, responsible for COVID-19, caused a series of atypical respiratory diseases in the city of Wuhan, China.^[1]^ Coronaviruses (CoVs) are singlestranded ribonucleic acid viruses that are zoonotic in nature^[2]^ and are divided into 4 genera: α-/β-/γ-/δ-CoV, where α- and β-CoV are capable of infecting mammals.^[3-5]^ Six types of CoVs have already been identified as viruses susceptible to humans, among which α-CoVs: HcoV-229E, HcoV-NL63, and β-CoVs: HCoV-HKU1 and HcoV-OC43 have low pathogenicity, and the other 2 known as β-CoVs, SARS-CoV and MERS-CoV, lead to serious respiratory tract infections and are potentially fatal.^[5,6]^

The SARS-CoV-2 virus is highly transmissible among humans, and despite the use of public health initiatives to combat the pandemic, there was an accelerated spread of the pandemic,^[7,8]^ leading to the death nearly 6 million people worldwide by early February 2022.^[9]^ COVID-19 is a syndrome that initially affects the respiratory system and can progress to atypical pneumonia. SARS-CoV-2 enters cells via angiotensin-converting enzyme 2 (ACE2) receptors, which are widely expressed in the pulmonary endothelium.^[10]^ As the disease progresses, the virus affects other important systems, such as cardiovascular, gastrointestinal, renal, and skin.^[11-14]^ COVID-19 has been shown to be a disease of prolonged evolution, requiring hospitalization in the intensive care unit (ICU) for long periods, which can lead to the development of physical and functional sequelae.^[15]^ In this sense, a common problem resulting from severe systemic infection of COVID-19 and its clinical deterioration is the development of pressure injury (PI) in critically ill patients.^[16]^

PI is defined as localized damage to the skin or underlying tissues, usually on a bony prominence, which may also be related to the use of medical devices. Injury usually occurs as a result of intense and/or prolonged pressure in combination with shearing and can affect several areas of the body.^[17,18]^ In a systematic review published in 2018, the cumulative incidence of PI in the ICU varied between 6.6% and 36.8%.^[19]^ In Brazil, some studies report that the incidence in the ICU varies between 17.2% and 41.0%.^[20-22]^

The development of a PI is a burden for the patient and the health system, as it increases the length of hospital stay and the risk of morbidity and mortality, decreases the quality of life, and substantially increases the costs for health systems.^[23-27]^ In the USA, the cost of treating PI acquired during hospitalization exceeds US$ 26.8 billion.^[24]^ As a result, health insurance from the Center for Medicare and Medicaid Services, USA no longer reimburses the excess costs of PI acquired during hospitalization since 2008,^[28]^ with hospitals responsible for this demand.

The pathophysiology of PI shows that vasoconstriction associated with impaired oxygen transport capacity is also responsible for a lower tolerance of the tissue to pressure,^[17]^ especially in critical patients who have immobility and reduced perfusion, which are also characteristic of patients with COVID-19 in critical condition.^[29]^ COVID-19-related PI prevalence data are incipient; however, researchers have observed an increase in PI cases in critically ill patients during the pandemic. A study of 74 patients confirmed for COVID-19, under invasive mechanical ventilation and treated with pronation therapy in ICUs in Madrid, showed a 77% prevalence of PI.^[30]^ In New York City, in a large health care center, the prevalence of PI in critically ill patients with COVID-19 was 3 times higher than in critically ill patients without COVID-19. Higher rates of PI were recorded in patients requiring prone positioning, as well as unusual presentations, multiplicity of occurrences, observed speed of onset, and severity of injury.^[31,32]^

The data mentioned emerge some questions: is the presence of systemic factors (caused by a biological agent, the SARS-CoV-2 virus) and local factors (caused by external mechanical load) a coincidence that, by specific mechanisms, can cause vascular, tissue, and ischemic damage? Does COVID-19 infection increase the risk of PI in critically ill patients?

We found no studies that addressed the physiological evidence that could explain the increased risk of developing PI in critically ill patients with COVID-19. However, prevalence studies have shown a substantial increase in PI in critically ill patients with COVID-19. In this regard, understanding the physiological changes that contribute to the increased risk of developing PI in critically ill patients with COVID-19 may lead to specific and systematic prevention measures in these patients, which would also fill the knowledge gap in this area.

The aim of this study was to perform a narrative review on the physiological evidence for increased risk in the development of pressure injuries in critically ill patients with COVID-19.

## 2. Method

This is a narrative review on studies of the physiology of COVID-19 and PI (population with no age limit) that excluded articles not written in English.

We searched the following databases: Public Medline (PubMed), Scientific Electronic Library Online (SciELO), and Google Scholar with these descriptors: “COVID-19”, “SARS-CoV-2”, “Pressure Ulcer”, “Pressure Injury”, “Cytokines”, “Blood Coagulation”, and “Ischemia”. The articles published between January 1, 2020 to December 31, 2020 that were selected for reading specifically addressed the object of the present study and were discussed in this review. Research Ethics Committee approval was not required because this is a study based on data published in other scientific articles.

## 3. Results

The initial search detected 1359 articles. Of these, 55 were selected and discussed in this review, and addressed the physiological evidence of increased risk of PI development in critically ill patients with COVID-19.

## 4. Discussion

### 4.1. Physiological, cellular, and molecular changes caused by the biological agent SARS-CoV-2

Infection with COVID-19 manifests itself in 2 phases: the first is the endogenous immune response, which is related to the patient’s innate response, in which the activation of the immune system is believed to be linked to the specific human leukocyte antigen and to the patient’s histocompatibility complex.^[33]^ In the first phase, the aim is to destroy the virus through cytokines. The second phase of the immune response consists of the activation of cytokines, but this time in excess, leading to the formation of a “cytokine storm,” which occurs mainly in the respiratory epithelium^[34]^ with hyperexpression of interleukin-1beta (IL-1beta), interleukin-6 (IL-6), and alpha tumor necrosis factor (TNF-alpha), which can lead to damage to the pulmonary microvasculature and, consequently, affect apoptosis and chemotaxis, decrease epithelial barriers, and cause alveolar edema.^[35]^ The cytokine storm in COVID-19 is associated with the development and progression of acute respiratory syndrome, in addition to being associated with higher levels of mortality and also with the damage and failure of extrapulmonary tissues and organs.^[34]^

A striking feature of patients who develop severe COVID-19 is the prevalence of specific forms of vasculopathy, thrombotic microangiopathy, and intravascular coagulopathy, as suggested by clinical evidence.^[36,37]^ It is inferred that this condition is due to the direct link between hemostasis and the inflammatory and immunological responses, suggesting that SARS- CoV-2 impairs innate and adaptive antiviral responses, triggering hyperinflammation and deregulating the renin-angiotensin-aldosterone system (RAAS).^[38]^ Thus, acute lung injury can lead to hypoxemia. Hyperinflammation, RAAS, and hypoxemia induce endothelial dysfunction and a hypercoagulable state, leading to systemic immunothrombosis, which can consequently cause generalized damage.^[36]^

This state of hyperinflammation is related to the ability of SARS-CoV-1, due to the expression of specific proteins, has to inhibit the production of type I interferon through the inhibition of the toll-like receptor 3 signaling pathways and toll-like receptor 7, for example,^[39-41]^ which is fundamental in the host’s innate response against viral infections. Thus, the antiviral response is delayed, facilitating the viral replication process as well as the extensive direct cytopathic effects induced by the virus in the early stages of the disease.^[42]^ SARS-CoV-2 is believed to have this same property, since the 2 viral forms have approximately 85% homology.^[38]^ The delayed activity of type I interferon, in conjunction with cytokines, chemokines, and molecular patterns associated with damage released by infected pneumocytes, may be associated with excessive infiltration of pathogenic inflammatory monocytes and neutrophils in the lung parenchyma, as it promotes increased vascular permeability.^[42]^ Thus, the production of high levels of pro-inflammatory cytokines is induced, with emphasis on IL-1beta, IL-6, TNF-alpha, and chemokines, which can culminate in hyperinflammation and the cytokine storm, which characterizes the most severe COVID-19, in addition to promoting upregulation of procoagulants.^[36,43]^ Due to the high expression of cytokines, with emphasis on IL-1beta and TNF-alpha, there is a promotion of endothelial activation and dysfunction, contributing to the manifestation of a prothrombotic state since it results in an increase in vascular permeability that is associated with excess generation of thrombin, an important platelet activator, and inhibition of fibrinolysis, resulting from the systemic effects of inflammation.^[36,44,45]^

During infection, platelets, clotting factors, and effector systems of the innate immune system interact to form clots in a process called immunothrombosis^[46-48]^ to inhibit the spread of the pathogen. However, when this process becomes widespread and uncontrolled, it can result in potentially devastating microangiopathy.^[44]^ This is mainly due to the imbalance of plasminogen activator inhibitor 1 (PAI-1), which tends to be elevated in severe cases of acute respiratory distress syndrome (ARDS) associated with SARS-CoV, indicating a hypofibrinolytic state associated with the pro-state coagulant, resulting in relative hypofibrinolysis, leading to fibrin deposition in the alveoli and perialveolar capillary microthrombosis.^[49]^ Thus, there may be a progression to ARDS. In addition, since the endothelium actively expresses ACE2 and has been shown to be an active site of SARS-CoV-1 infection, it is possible that SARS-CoV-2 can directly infect these cells.^[50]^ However, the difference in the infection of the different micro and macrovascular beds and between the organs is justified, since, as the entry of the virus into the cells occurs mainly through endocytosis,^[51,52]^ it will strongly depend on the expression in each ACE2 tissue and the availability of transmembrane protease serine 2 protein or other proteases to cleave the viral peak.^[53]^

It should also be noted that the RAAS system is intrinsically linked to the coagulation cascade, which, associated with ACE2 dysfunction caused by COVID-19 infection, can induce a hypertensive condition,^[54,55]^ possibly exacerbating the processes of immunothrombosis and driving an even more significant formation of microthrombi in infected patients.^[36]^ This occurs because ACE2 antagonizes the expression of angiotensin 2, decreasing its vasoconstrictor, pro-inflammatory, pro-apoptotic, pro-thrombotic, mitogenic, and metabolic effects, among others.^[54]^ Thus, the expression of tissue factor and PAI-1 is induced by endothelial cells through the angiotensin type 1 receptor, contributing to an imbalance of PAI-1/tissue plasminogen activator in a hypercoagulable state.^[56,57]^ In addition, the hypercoagulable state can potentially be increased by other clinical factors, including hypoxemia secondary to acute lung injury, hyperthermia, which can activate platelets and coagulation or hypovolemia secondary to loss of gastrointestinal fluids or negative water balance.^[58]^ Hypoxemia causes increased expression of hypoxia-inducible factors, increasing the inflammatory condition in a way that increases blood viscosity and aggravates hypercoagulability. In addition, hypoxia-inducible factors can directly activate platelets and clotting factors, increasing the expression of tissue factor, increasing PAI-1, and inhibiting endogenous anticoagulant protein S.^[59]^

### 4.2. Pathophysiology of PI

The detection of a PI is evidenced by visual and tactile changes on the skin surface, but physiological changes below the skin may precede changes on the surface.^[60]^ In this sense, immobility, skin status, and decreased perfusion are considered direct causal factors in the development of PI. There is strong scientific epidemiological evidence that low perfusion and skin status reduce tissue tolerance to pressure and increase the likelihood of developing PI, and these factors are clearly involved in the susceptibility and tolerance of the individual’s structure. As a result, the status of the skin corresponds to the individual geometry (morphology) of the bones and soft tissues, mechanical properties of the tissues, thermal and transport properties, and physiological aspects of tissue restoration. Perfusion, on the other hand, corresponds to individual transport, thermal properties, and the element of physiology and structure repair, and is related to factors that impair circulation. In addition to factors such as nutrient delivery and waste removal, oxygen transport capacity is important in maintaining healthy tissues.^[18]^

Coleman et al^[18]^ argue that the absence of risk factors on the susceptibility and individual tolerance side or in the mechanical boundary conditions of the bone structure affects the likelihood of developing PI; that is, a patient with good perfusion can withstand higher levels of immobility without developing PI than those with poor perfusion. On the other hand, prolonged pressure and/or shear that are not relieved leads to ischemia when external tissue compression exceeds capillary pressure^[61,62]^ and may be related to reperfusion injury.^[60]^

Deformation of tissues subjected to compression leads to lymphatic dysfunction, on the other hand, ischemia leads to oxidative stress and hypoxia, which can contribute to nutrient depletion, accumulation of toxic metabolites, and local inflammatory changes. Hypoxia and increased vascular permeability contribute to apoptosis and necrosis in tissues and, consequently, accumulation of interstitial fluid and damage to subepidermal tissues, which can manifest themselves in visible and tactile changes in the skin and progress to PI stage 4.^[60]^

After an initial injury, endothelial injury, due to mechanical tissue compression in an immobile patient, the need for hemostasis arises. To this end, the components of the extracellular matrix (ECM) bind and activate circulating platelets, which undergo adhesion and aggregation. Damaged tissue and aggregated platelets trigger extrinsic and intrinsic coagulation pathways, consolidating the fibrin-platelet clot.^[63]^ ECM occupies the space between cells, being formed by proteins and polysaccharides in water, providing a means by which nutrients and residues can be transported from one cell to another^[64]^; it is an essential component in the healing process^[65]^ and is a reservoir for cytokines and growth factors.^[63]^ However, with the maintenance of endothelial damage due to compression, the accumulation of fluids in the extracellular space can result from changes in hydrostatic or oncotic pressure in the microvascular walls, changes in the endothelial walls of cells, or changes in the lymphatic system. In addition, the accumulation of interstitial fluid may also be the result of inflammatory mediators.^[60,66]^

Inflammation as a response to injury - tissue relief not relieved - includes the release of inflammatory mediators responsible for the permeability of microvessels, vasodilation, and leukocyte recruitment, which results in the release of reactive oxygen and nitrogen species that degrade the ECM in order to decrease the pressure of the interstitial fluid.^[60,66]^ Inflammation involves tissue degradation and cleaning of cellular and extracellular fragments and pathogens.^[63]^ Neutrophils are the first inflammatory cells to respond to mediators and start by adhering to the cell walls of the vascular endothelium of the injured tissue. Next, they release elastase and collagenase, which facilitate their migration through the basement membrane surrounding the endothelial cells and the transition to ECM at the injury site. Neutrophils also produce and release inflammatory mediators, such as TNF-alpha and interleukin-1, which recruit and activate even more fibroblasts and epithelial cells.^[65]^

However, in the presence of a harmful external stimulus that causes tissue damage, the inflammatory process can still increase and prolong.^[63]^ Overproduction or prolonged expression of TNF-alpha in the inflammatory phase can cause greater tissue destruction due to the overactivation of immune cells and the production of proteases, together with decreased collagen synthesis and reduced granulation tissue formation.^[67,68]^ Neutrophils produce and contain high levels of destructive proteases and oxygen free radicals, which are used to digest phagocyte materials. When these cells die, these substances are released in the local area of the lesion, which makes the environment favorable for extensive tissue damage and durability of the inflammatory phase.^[65]^ Furthermore, it is known that TNF-alpha in action with IL-1beta and IL-6 causes local inflammation^[69,70]^; therefore, in a compressed area, there is a significant increase in the expression of genes associated with inflammation, contributing to the formation of PI.^[71]^

In the formation of PI, the role of interleukin-10 (IL-10) and tissue inhibitor of metalloproteinase 1 as inflammation inhibitors^[70,72-74]^ has already been established; however, in tests carried out in a pre-clinical study, the upregulated levels of these genes were lower than those of inflammatory cytokines, such as interleukin-1alpha, IL-1 beta, IL-6, and TNF-alpha. Thus, edema and erythema occurred despite the presence of IL-10 and tissue inhibitor of metalloproteinase 1. Thus, interleukin-1alpha, IL-1beta, IL-6, and TNF-alpha trigger or increase inflammation,^[69]^ and these pro-inflammatory cytokines increased markedly more than those that inhibit inflammation. ELISA data showed a small increase in IL-1beta and IL-6 and a decrease in IL-10, suggesting an imbalance of inflammatory cytokines and anti-inflammatory cytokines, since IL-1beta and IL-6 increased in preference to other cytokines, which may indicate a more prominent role for IL-1beta and IL-6 in the formation of PI.^[71]^

### 4.3. COVID-19 and PI

The main function of red blood cells is to transport hemoglobin that carries oxygen from the lungs to the tissues of the body. The number of red blood cells in the circulatory system is regulated within very narrow limits, so that there is always an adequate number of cells to provide adequate tissue oxygenation, without interfering with blood flow to the tissues.^[75]^ An observational study examined the relationship between oxygenation and tissue perfusion in the development of PI in 30 critically ill patients receiving mechanical ventilation. The results showed that mechanically ventilated patients who developed PI had significantly lower diastolic blood pressure values than patients who remained without PI,^[76]^ corroborating the findings of other researchers who reported the importance of good blood perfusion to improve tolerance pressure tissues.^[18,77]^

A study evaluated the functioning of capillaries in muscle tissue of animal models that received load pressures of 12, 37, and 78 kPa directly in the gracilis muscle for 2 hours. The temperatures of the intervention and control muscles were recorded using thermography as a measure of ischemia level. A non-linear finite element model of muscle-fascicle deformation was subjected to pressures of 12 to 120 kPa with and without simultaneous shear stress of up to 8%.

The results showed that physiological pressures in the range of 12 to 78kPa can cause partial ischemia in skeletal muscle tissue after 10 minutes and complete ischemia after 40 minutes, possibly due to blood vessel blockage by clots, observed in the histology of the muscles. However, the greater the magnitude of the shear deformation added simultaneously to the pressure, the faster the decrease in the capillary open section area, with a 45% greater decrease in the capillary area than when only pressures up to 120 kPa were applied. In addition, the study showed that the stained slides of animals exposed to non-zero pressures showed extensive muscle cell death, evident from substantial loss of cross striation, while the unloaded muscles showed normal cross striation. Thermography and histological studies suggest that the generalized death of muscle cells is caused by a period of less than 40 minutes of pressure.^[78]^

An observational study carried out with elderly patients investigated the extent to which internal risk factors for the development of PI were related to the blood flow response after the relief of a test pressure load. The results showed that the presence of cardiovascular disease and stroke showed a statistically significant relationship with impaired blood flow response. The delayed latency found showed a similarity with the so-called “non-reperfusion phenomenon”, which occurs after periods of ischemia and has also been described in connection with ischemia-reperfusion conditions.^[79]^ As a result of this phenomenon, it is believed to result in swelling of the capillary endothelium, increased peripheral vascular hydraulic resistance, and impaired microvascular blood flow. Oxygen free radicals involved in the endothelial damage process are also observed, in addition to pro-inflammatory substances and toxic metabolites, considering the ischemia-reperfusion process as a major local inflammatory reaction that influences the blood flow response.^[79,80]^

The ARDS that accompanies the serious disease of COVID-19 involves severe hypoxemia, high respiratory compliance, and high shunt fraction, determining the need for prolonged mechanical ventilation.^[81,82]^ The disease also demonstrates the systemic characteristics of a hypercoagulable state, unlike classical ARDS. In addition, a pattern of tissue damage consistent with complement-mediated microvascular injury was observed in the lungs and skin of individuals with severe COVID-19.^[83]^

Magro et al,^[83]^ from the analysis of pulmonary and cutaneous biopsy and autopsy of 5 patients with severe COVID-19, demonstrated that patients with severe SARS-CoV-2 are more predisposed to the development of vascular injury in other sites, in addition to the lung, due to the activation mechanisms of the lectin complement pathway. This is because one of the SARS-CoV-2 glycoproteins binds to mannose-binding lectin (MBL), forming an MBL complex with mannose-binding protein-associated serine protease 2 (MASP-2) that activates the alternative complement pathway (AP).^[84]^ The activation of AP triggers inflammation, simultaneous activation of the coagulation cascade,^[85,86]^ and causes changes, such as thrombosis and complement microvascular deposits.^[83]^ The activation of AP is almost immediate because MBL has a domain for the recognition of carbohydrates by which it joins with mannoses that are exposed on the surface of pathogens. When they are linked to the pathogen, the mannose-binding protein-associated serine protease 1 and MASP-2 activate to break complement component 4 and complement component 2 and create complement component 3 convertase to continue the complement pathway to activate complement component 5 and start the membrane attack complex, producing a local inflammatory process. It is also known that MASP-2 activates prothrombin, favoring the formation of clots.^[87]^

Magro et al^[83]^ also observed 5 critically ill patients with COVID-19, characterized by respiratory failure and purple skin rash. The patients had a hypercoagulable state, with prolonged prothrombin time, elevated levels of D-dimer and fibrinogen, and activated partial thromboplastin time close to normal. Lung autopsies, skin biopsies, and clinical, microscopic, and immunohistochemical analyses were performed. COVID-19 pneumonitis was found, showing a pauciinflammatory septal capillary lesion with significant fibrin deposition in the alveolar spaces and interstitial and intra-alveolar accumulation of neutrophils. The pulmonary findings were accompanied by significant deposits of components of the complement complement membrane attack complex (membrane attack complex), complement component 4d, and serine protease associated with MBL and MASP-2 in the microvasculature, including deposits in areas of normal-looking lung, consistent with sustained systemic activation of complement pathways. Similarly, purpuric skin lesions showed pauciinflammatory thrombogenic vasculopathy with deposition of complement complement membrane attack complex and complement component 4d on the skin involved with the lesion and on normal-looking skin. Therefore, a pattern of tissue damage consistent with complement-mediated microvascular injury was observed in the lungs and/or skin of the individuals evaluated.

Based on these findings, international bodies recommend that discolored areas in any body area subjected to pressure or shear loading be evaluated to detect differences in tissue consistency and temperature to discard PI; theoretically, the same thrombogenic vascular changes related to COVID-19 that they occur in the skin may occur in the underlying soft tissue, the muscle, making these tissues less tolerant to the damaging effects of pressure and shear.^[88,89]^ This factor relationship is shown in Figure 1.

**Figure:**
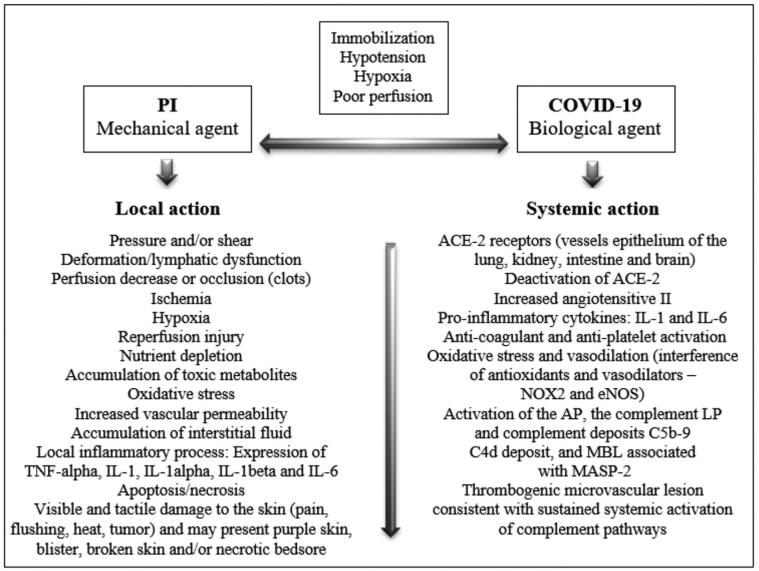
**Figure 1.** Microvascular damage caused by a cascade of biological agents versus mechanical agents, both could occur independent or simultaneously in a patient with COVID-19 at the ICU. ACE-2 = angiotensin converting enzyme 2, AP = alternative complement pathway, C4d = complement component 4d, C5b-9 = complement membrane attack complex, eNOS = endothelial nitric oxide sintetase, ICU = intensive care unit, IL-1 = interleukin-1, IL-1alpha = interleukin-1alpha, IL-1beta = interleukin-1beta, IL-6 = interleukin-6, LP = lectin complement pathway, MASP-2 = mannose-binding protein-associated serine protease 2, MBL = mannose-binding lectin, NOX2 = NADPH oxidase 2, TNF-alpha = tumor necrosis factor-alpha.

Immobile, hypotensive, hypoxic patients with reduced perfusion have a high risk of developing PI in regions that support pressure and/or shear and are also the characteristics of patients with critical COVID-19.^[88,89]^

### 4.4. Limitations

The present narrative review presents a theoretical basis for the increased risk that critically ill patients with COVID-19 have of developing PI. The hypothesis and its theory should be tested in clinical trials with ICU patients, with the study consisting of an intervention group (patients confirmed with COVID-19) versus a control group (as a benchmark for comparisons between groups). Participants could be classified according to the disease severity (COVID-19) and followed longitudinally regarding the occurrence of PI, number of PIs, site of highest occurrence, observed onset speed, severity of injury, and risk of death.

The focus of this review stopped at physiological aspects in the development of PI in patients with COVID-19. Aspects such as personal, professional, and institutional barriers of healthcare teams in assisting ICU patients were not surveyed, which may also contribute to the increased incidence of PI during the pandemic. Thus, the analysis of this review may be an important and well justified starting point to establish an agenda for the investigations focused on understanding the pathophysiological basis that circumscribe to the increased risk of critically ill patients with COVID-19 to develop PI.

## 5. Conclusion

This narrative review presents a rational basis to understand the physiological evidence for the increased risk of developing PI in critically ill patients with COVID-19. These patients with COVID-19 generally have low blood oxygen concentrations. In addition to having a systemically enhanced inflammatory system, many develop systemic thrombogenic microvascular injury by sustained activation of the complement system, compromising the flow and adequate blood perfusion for body tissues and organs, such as the skin.

Given the complexity of COVID-19 and PI, it is possible to notice a coincidence in the presence of systemic factors (caused by a biological agent, the SARS-CoV-2 virus) and local factors (caused by external mechanical load) related to immobility, hypotension, hypoxia, and reduced perfusion, which by specific mechanisms can cause vascular damage, ischemia, and damage to organs and/or tissues. Therefore, unlike typical SARS and other diseases and comorbidities that commonly lead the patient to ICU admission and treatment, such as high blood pressure, stroke, heart attack, cancer, tuberculosis, COVID-19, and systemic viral spread in critical patients, show that systemic and local factors overlap.

## Acknowledgments

This study was supported by a scholarship from the Coordination of Improvement of Higher Level Personnel Brazil (CAPES).

## Author contributions

**Conceptualization:** Miriam Viviane Baron, Bartira Ercília Pinheiro da Costa.

**Formal analysis:** Cristine Brandenburg, Gabriela Feltez, Aline Ronis Sampaio, Marcus Vinicius de Mello Pinto, Sonia Carvalho, Patrícia Froes Meyer, Felice Picariello, Esteban Fortuny Pacheco, Isabel Cristina Reinheimer, Alexandre Gomes Sancho.

**Investigation:** Bartira Ercília Pinheiro da Costa, Michele Paula dos Santos, Tais Michele Werle, Gabriela D. L. G. Scherer, Mariana Martins Dantas Santos, Luis Manuel Ley Dominguez.

**Methodology:** Miriam Viviane Baron, Cristine Brandenburg, Gabriela Feltez, Aline Ronis Sampaio, Marcus Vinicius de Mello Pinto, Sonia Carvalho, Patrícia Froes Meyer, Felice Picariello, Esteban Fortuny Pacheco, Isabel Cristina Reinheimer, Alexandre Gomes Sancho.

**Supervision:** Cristine Brandenburg, Gabriela Feltez, Aline Ronis Sampaio, Marcus Vinicius de Mello Pinto, Sonia Carvalho, Patrícia Froes Meyer, Felice Picariello, Esteban Fortuny Pacheco, Isabel Cristina Reinheimer, Alexandre Gomes Sancho.

**Visualization:** Michele Paula dos Santos, Tais Michele Werle, Gabriela D. L. G. Scherer, Mariana Martins Dantas Santos, Luis Manuel Ley Dominguez, Bartira Ercília Pinheiro da Costa.

**Writing - original draft:** Miriam Viviane Baron, Michele Paula dos Santos, Tais Michele Werle, Gabriela D. L. G. Scherer, Mariana Martins Dantas Santos, Luis Manuel Ley Dominguez.

**Writing - review & editing:** Miriam Viviane Baron, Michele Paula dos Santos, Tais Michele Werle, Gabriela D. L. G. Scherer, Mariana Martins Dantas Santos, Luis Manuel Ley Dominguez, Cristine Brandenburg, Gabriela Feltez, Aline Ronis Sampaio, Marcus Vinicius de Mello Pinto, Sonia Carvalho, Patrícia Froes Meyer, Felice Picariello, Esteban Fortuny Pacheco, Isabel Cristina Reinheimer, Alexandre Gomes Sancho, Bartira Ercília Pinheiro da Costa.
